# Further Exploration of Sucrose–Citric Acid Adhesive: Investigation of Optimal Hot-Pressing Conditions for Plywood and Curing Behavior

**DOI:** 10.3390/polym11121996

**Published:** 2019-12-02

**Authors:** Zhongyuan Zhao, Shunsuke Sakai, Di Wu, Zhen Chen, Nan Zhu, Caoxing Huang, Shijing Sun, Min Zhang, Kenji Umemura, Qiang Yong

**Affiliations:** 1 College of Furnishings and Industrial Design, Nanjing Forestry University, Nanjing 210037, China; nfuwudi@163.com; 2 Laboratory of Sustainable Materials, Research Institute for Sustainable Humanosphere, Kyoto University, Gokasho, Uji, Kyoto 611-0011, Japan; shunsuke_sakai@rish.kyoto-u.ac.jp (S.S.); zhang888@rish.kyoto-u.ac.jp (M.Z.); 3 Qingdao Institute of Bioenergy and Bioprocess Technology, Chinese Academy of Sciences, Qingdao 266101, China; chenzhen@qibebt.ac.cn; 4 Jiangsu Center of Supervision & Testing on Green Degradable Material Quality, Nanjing 210019, China; nzhuwork@126.com; 5 College of Chemical Engineering, Nanjing Forestry University, Nanjing 210037, China; szxapy@163.com; 6 College of Material Science and Engineering, Nanjing Forestry University, Nanjing 210037, China; sunsj-611@163.com

**Keywords:** eco-friendly adhesive, sucrose, citric acid, plywood

## Abstract

In previous research, sucrose and citric acid were used to synthesize an eco-friendly plywood adhesive. Herein, further research was performed to determine the optimal hot-pressing conditions and curing behavior of a sucrose-citric acid (SC) adhesive. The results of dry and wet shear strength measurements showed that the optimal hot-pressing temperature, hot-pressing time, and spread rate of plywood samples bonded by the SC adhesive were 190 °C, 7 min, and 140 g/m^2^, respectively. When plywood was bonded at the optimal hot-pressing conditions, the wet shear strength met the requirements of the China National Standard GB/T 9846-2015. Thermal analysis showed that the thermal degradation and endothermic reaction temperatures of the SC 25/75 adhesive were lower than either sucrose or citric acid individually. In addition, the insoluble mass proportion increased with the heating temperature and time. The Pyrolysis Gas Chromatography and Mass Spectrometr (Py-GC/MS) analysis confirmed that the SC adhesive was cured by the reaction between furan compounds, saccharide, and citric acid, and the resulting polymer appeared to be joined by ether linkages.

## 1. Introduction

As fossil resources are decreasing, the development of bio-based materials has attracted increasing attention [1,2,3,4,5], and these novel materials are expected to be utilized in many fields. However, most current bio-based materials used for construction, furniture, and flooring are glued wood-based materials, such as particleboards, plywood and fibreboards [6,7], for construction, furniture and floorings [8,9]. Most of these materials are fabricated using synthetic adhesives that use raw materials derived from fossil fuel-based resources [10,11], many of which also contain formaldehyde to ensure sufficient reactivity and adhesive performance [12,13]. Due to the rapidly increasing environmental awareness and the toxicity of formaldehyde, some methods have been applied to reduce formaldehyde emissions, such as lowering the molar ratio of formaldehyde in the Urea-formaldehyde (UF) resin, adding formaldehyde scavengers in the resin, post-treatment with barrier layers and the development of green and sustainable alternative wood adhesives based on renewable resources [14,15,16,17,18,19].

Recently, it was found that citric acid can be utilized as an eco-friendly adhesive for wood-based materials by spraying an aqueous citric acid solution on wood particles [20,21,22]. After or without prior drying, the sprayed wood particles are hot pressed at 180–200 °C to fabricate particleboards. The mechanical properties and water resistance of the resulting particleboards satisfied the JIS A 5908 standard, and the proposed reaction mechanism involves the formation of carbonyl groups between citric acid and wood components [22]. Sucrose has also been added to the citric acid solution to promote bonding [23,24]. However, although this citric acid–sucrose adhesive can be used to manufacture particleboard, it is difficult to produce plywood with due to the fact of its low viscosity and low solid content. To overcome this limitation, a synthetic method was developed to obtain a novel citric acid–sucrose (SC) adhesive with an appropriate viscosity, high solid content and good bond performance which can be utilized to manufacture plywood.

Previous research [25] showed that the optimal mass proportion between sucrose and citric acid is 25/75, the synthesis temperature is 100 °C and the synthesis time is 2 h. In addition, the synthesis mechanism was studied by both ^13^C NMR analysis and HPLC, and the chemical composition manifesting caramelization occurred during synthesis. The results of ATR FTIR indicated the formation of a furan ring and carbonyl and ether groups in the cured insoluble mass of the SC adhesive [25]. In this study, to determine the optimal hot-pressing conditions, the effects of the hot-pressing temperature, hot-pressing time and spread rate on the bonding performance of plywood bonded by SC adhesive were investigated. Furthermore, the curing behaviour of this novel adhesive was confirmed by thermal analysis, measuring the insoluble mass proportion, pyrolysis gas chromatography and mass spectrometry

## 2. Materials and Methods

### 2.1. Materials

Sucrose (analytical grade) and citric acid (analytical grade) were purchased from Sinopharm Chemical Reagent Co., Ltd. (Shanghai, China). Each reagent was vacuum dried at 60 °C until reaching a constant mass prior to use in experiments. Poplar veneers (species: *Populus tomentosa* Carr) were purchased from Zuogezhuang, Langfang, China, and the product method was rotary cutting.

### 2.2. Preparation of Sucrose–Citric Acid (SC) Adhesives

Previous research showed that the optimal mass ratio of sucrose and citric acid is 25/75, the synthesis temperature is 100 °C and the synthesis time is 2 h. Sucrose and citric acid were mixed at a mass proportion of 25/75 and poured into a three-neck flask with distilled water to synthesize SC adhesives with 80 wt % solid content. The mixture was then heated in an oil bath at 100 °C for 2 h and sheared at 180 rpm/min to facilitate synthesis of the sucrose–citric acid (SC) adhesive. The pH of the adhesive was measured at 30 °C using a Leici pH meter PHBJ-206 (Leici, Shanghai, China), and the viscosity of the adhesive was measured using a HAAKE rotational rheometer MA S60 (HAAKE CO., Karlsruhe, Germany). The pH of the SC adhesive was 0.9 and its viscosity was 640 mPa·s. Sucrose and citric acid were also treated using the synthetic route above, and the obtained solution was marked as SC 100/0 and SC 0/100 as controls. Synthesized SC adhesive was sealed and stored at room temperature for at least 3 days until further use.

### 2.3. Bond Performance

#### 2.3.1. Manufacture of Plywood

Synthesized SC adhesives were used to manufacture a three-layer plywood sample (300 mm × 300 mm), the bond performance of which was evaluated. The moisture content and thickness of the veneers were 9.8–11% and 1.5 mm, respectively. The SC adhesives were applied to the core veneer at a spread rate of 140 g/m^2^ for a single veneer surface. The coated veneer was stacked between two uncoated veneers so that the grain directions of both adjacent veneers were perpendicular to each other. All assembled three-layered plywood samples with SC adhesive were hot-pressed at three manufacturing conditions to investigate the effects of hot-pressing temperature, hot-pressing time and spread rate on the bond performance; in total, 12 plywood pieces were produced. The details of these conditions are shown in Table 1.

#### 2.3.2. Shear Strength Measurement

The prepared plywood samples were cut into standard tensile shear test specimens according to China National Standards (GB/T 9846.7-2004). Twelve plywood specimens (10 × 25 mm) were cut from each manufactured plywood sample, and six specimens were submerged in water at 63 ± 2 °C for 3 h. Then, the tensile shear strength of the plywood samples was measured under dry and wet conditions under a loading rate of 1.0 mm/min. Each plywood test was carried out in twelve replicates, and the average values, standard deviations and average wood failures were calculated. Statistical significance was considered for *p*-values < 0.05.

### 2.4. Curing Behaviour

#### 2.4.1. Thermal Analysis

The SC 25/75 adhesive and the control samples (SC 100/0 and SC0/100) were poured into separate glass vials and then frozen in a refrigerator. After samples were completely frozen, they were dried using a lyophilizer to obtain uncured samples which were analyzed by thermogravimetric analysis (TGA) and differential scanning calorimetry (DSC) using a Discovery TGA 55 (TA Instruments, Tokyo, Japan) and Discovery DSC 25 (TA Instruments, Tokyo, Japan), respectively. The samples were scanned from room temperature to 400 °C at a heating rate of 10 °C/min under nitrogen purging with flow rates of 70 mL/min and 50 mL/min, respectively.

#### 2.4.2. Insoluble Mass Proportion

Uncured SC adhesive was divided into 2 groups, and each of these groups was further separated into 4 parts. In Group 1, samples of uncured SC adhesive were heated at 130, 150, 170, and 190 °C for 7 min; in Group 2, samples were heated at 190 °C for 3, 5, 7, and 9 min to obtain cured adhesive samples. Approximately 2 g of each cured sample was then boiled in distilled water for 4 h to obtain an insoluble mass; this was carried out in triplicate. The obtained insoluble masses were vacuum dried at 60 °C until reaching a constant mass (15 h) and finally weighed. The insoluble mass proportion was calculated by the following equation:

(1) $$\mathrm{Insoluble}\mathrm{mass}\mathrm{proportion}\;\left(\%\right)=\frac{Weight\;of\;dried\;insoluble\;mass\;\left(g\right)}{Weight\;of\;heated\;sample\left(g\right)}\;\times 100\%$$

#### 2.4.3. Pyrolysis Gas Chromatography and Mass Spectrometry (Py-GC/MS)

Pyrolysis gas chromatography and mass spectrometry was used to investigate the chemical changes during and after curing. First, synthesized SC adhesives with sucrose/citric acid mass proportions of 100/0, 25/75, and 0/100 were lyophilized to obtained uncured adhesives. Volatile compounds produced during the curing were measured by a Py-GC/MS system (GCMS-QP2010, Shimadzu Co., Ltd., Kyoto, Japan). Approximately 1 mg samples were placed in a small cup and pyrolyzed at 170 °C for 60 s by a Multi-Shot pyrolyzer (EGA/PY-3030d, Frontier Laboratories, Ltd.), with a pyroprobe interface temperature of 220 °C and an Ultra ALLOY-5 capillary column (30 m × 0.25 mm i.d., 0.25 μm film thickness, Frontier Laboratories Ltd., Fukushima, Japan). The initial temperature of the column was set at 37 °C for 2 min, and then the temperature was increased to 100 °C at a rate of 30 °C /min. Once reaching 100 °C, the temperature was further increased to 220 °C at a rate of 15 °C/min and then held at 220 °C for 1 min. Then, to investigate the chemical composition of the cured adhesives, the insoluble mass of cured SC 25/75 was pyrolyzed at 500 °C for 60 s, and the pyroprobe interface temperature was set to 400 °C. The initial temperature of the column was held at 37 °C for 2 min, and then the temperature was increased to 100 °C at a rate of 30 °C/min. The temperature was immediately increased to 300 °C at a rate of 15 °C/min and held at 300 °C for 1 min. Mass spectrometry was operated in EI mode at 70 eV with an analysis range of 50–600 m/z and a scanning speed of 1250 amu/s. The pyrolysis products were identified using the NIST 08 mass spectral library, and the identified volatile components with the highest and >80 similarity index (SI) were recorded.

## 3. Results and Discussion

### 3.1. Effects of Hot-Pressing Conditions on the Bond Performance

**Figure 1:**
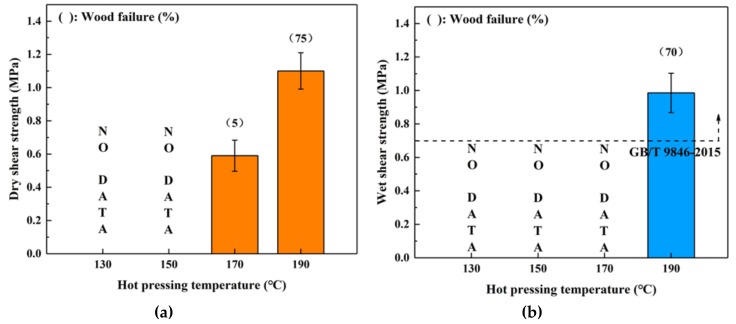
Effects of hot-pressing temperature on the bond performance of plywood: (**a**) dry shear strength, (**b**) wet shear strength.

To investigate the effects of different manufacturing conditions on the bonding properties, SC adhesive samples were used to fabricate plywood at various hot-pressing temperatures, hot-pressing times and spread rates. Figure 1 shows the dry and wet strength of plywood samples bonded from 130 to 190 °C. The boards cured at 130 and 150 °C showed no adhesion strength, and it seemed that the curing reaction did not occur at these temperatures. In contrast, a certain dry bond strength was obtained at hot-pressing temperatures higher than 170 °C, and the shear strength and wood failure strengths increased with increasing hot-pressing temperatures. However, only the board bonded at 190 °C maintained its original shape throughout the immersion treatment stage. This specimen also displayed a relatively higher wet shear strength (0.99 MPa) and wood failure (70%), indicating that the hot-pressing temperature of 190 °C led to sufficient adhesive curing. The wet shear strength of the plywood bonded by SC adhesive at 190 °C met the requirement of the China National Standard GB/T 9846-2015; thus, this hot-pressing temperature was determined to be the optimal manufacturing condition.

**Figure 2:**
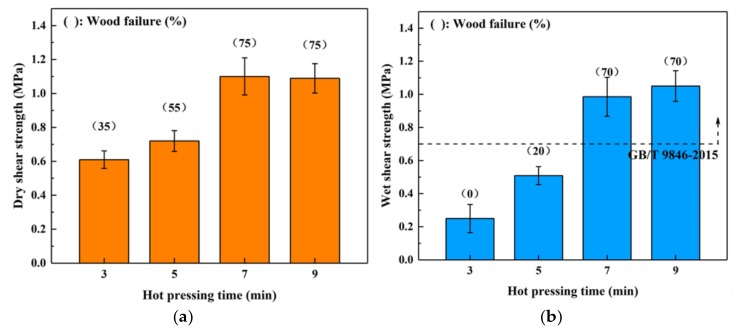
Effects of hot-pressing time on the bond performance of plywood: (**a**) dry shear strength, (**b**) wet shear strength.

The profiles visualized in Figure 2 show that both the dry and wet shear strengths of the boards bonded with SC adhesive exhibited a positive relationship with the hot-pressing time. For the dry shear strength, variance analysis (ANOVA) revealed no significant (*p* > 0.05) difference of the boards hot-pressed for 7 and 9 min which indicated that prolonging the hot-pressing time beyond 7 min would not significantly affect the bond strength of the SC adhesive. Regarding the wet shear strength, the water resistance of the samples bonded at 7 min or longer was greater than the amount set by the China National Standard GB/T 9846-2015. The maximum value was observed in the board bonded for 9 min (1.05 MPa). However, considering that the plywood bonded for 7 min also exhibited an excellent wet shear strength (0.99 MPa), the optimal hot-pressing time was considered as 7 min.

**Figure 3:**
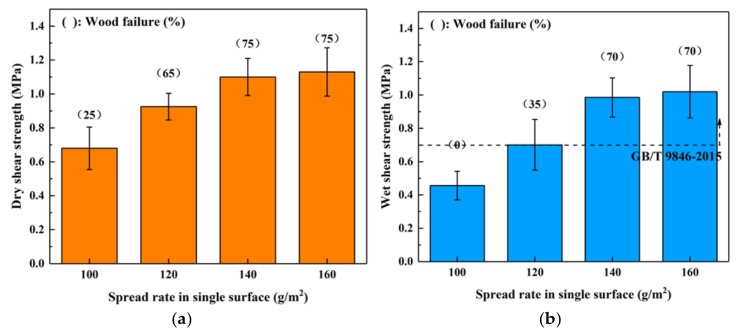
Effects of spread rate on the bond performance of plywood: (**a**) dry shear strength, (**b**) wet shear strength.

To investigate the influence of the spread rate on the bond performance of this high-solid content adhesive, the plywood samples were manufactured at 190 °C for 7 min with a 100–160 g/m^2^ spread rate on a single surface, and the results are shown in Figure 3. Both dry and wet shear strengths increased as the spread rate increased. When the spread rate was higher than 140 g/m^2^, the bond strength of SC adhesive plateaued, and the wood failure of dry and wet conditions remained at 70–75%. Thus, it appears that the SC adhesive achieved the highest bondability at this spread rate. In addition, the wet shear strength of the boards bonded using spread rates higher than 120 g/m^2^ satisfied the requirements of the China National Standard GB/T 9846-2015. Considering that the wet shear strength of the board bonded with a 140 g/m^2^ spread rate (0.99 MPa) already exceeded the standard requirement (0.7 MPa) by 41%, the optimal spread rate was determined to be 140 g/m^2^.

Based on the investigation of the bond performance above, the optimal manufacture conditions of SC adhesive were: hot-pressing temperature—190 °C; hot-pressing time—7 min; spread rate—140 g/m^2^. Although the properties of the plywood which were manufactured under the optimal hot-pressing conditions satisfied the requirements of the China National Standard GB/T 9846-2015, these fabrication conditions were harsher than the industrial conditions [7,14]. Therefore, the curing behavior and reaction mechanism should be clarified in this research, which is helpful in determining the method to use to reduce the manufacturing conditions in future research.

### 3.2. Curing Behaviour of SC Adhesive

#### 3.2.1. TG Analysis

**Figure 4:**
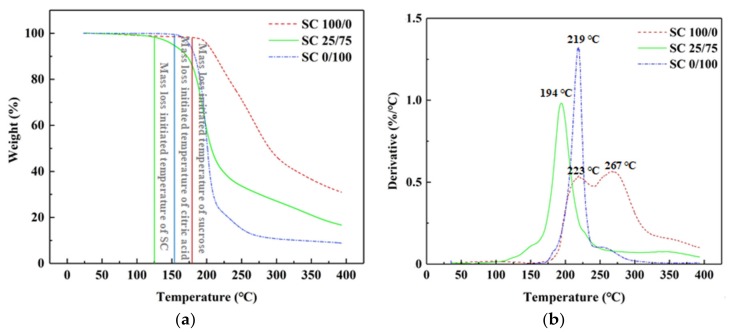
TG (**a**) and DTG (**b**) curves of sucrose (SC 100/0), citric acid (SC 0/100), and uncured SC adhesive (SC 25/75).

To clarify the curing behavior during heating treatment, thermal analyses were performed. Figure 4a shows the thermogravimetric (TG) curves of SC (25/75) adhesive, which served as a control, while Figure 4b shows the TG curves of freeze-dried sucrose (100/0) and citric acid (0/100) samples heated at 90 °C for 3 h with 80% solid content. The TG results show that sucrose exhibited a rapid mass loss beginning near 180 °C, possibly due to the thermal degradation of sucrose [26]. For citric acid, the thermal degradation began around 155 °C which was attributed to dehydration [27]. However, when analyzing SC 25/75, the preliminary weight loss occurred at a lower temperature (around 125 °C), possibly due to the citric acid-catalyzed thermal degradation of sucrose.

The DTG curves show that sucrose (SC 100/0) underwent a two-step degradation. The first step was observed at around 223 °C which was attributed to the caramelization of sucrose [28]. The second step of degradation occurred at 267 °C due to the formation of a black, aerated, and char-like solid [26]. For citric acid, a one-step thermal degradation was observed at approximately 219 °C which was attributed to the pyrolysis of citric acid during heating [27]. The onset of weight loss decreased to around 194 °C for SC 25/75 which was significantly lower than those of sucrose and citric acid only, demonstrating that citric acid could catalyze the thermal degradation of sucrose.

#### 3.2.2. DSC Analysis

**Figure 5:**
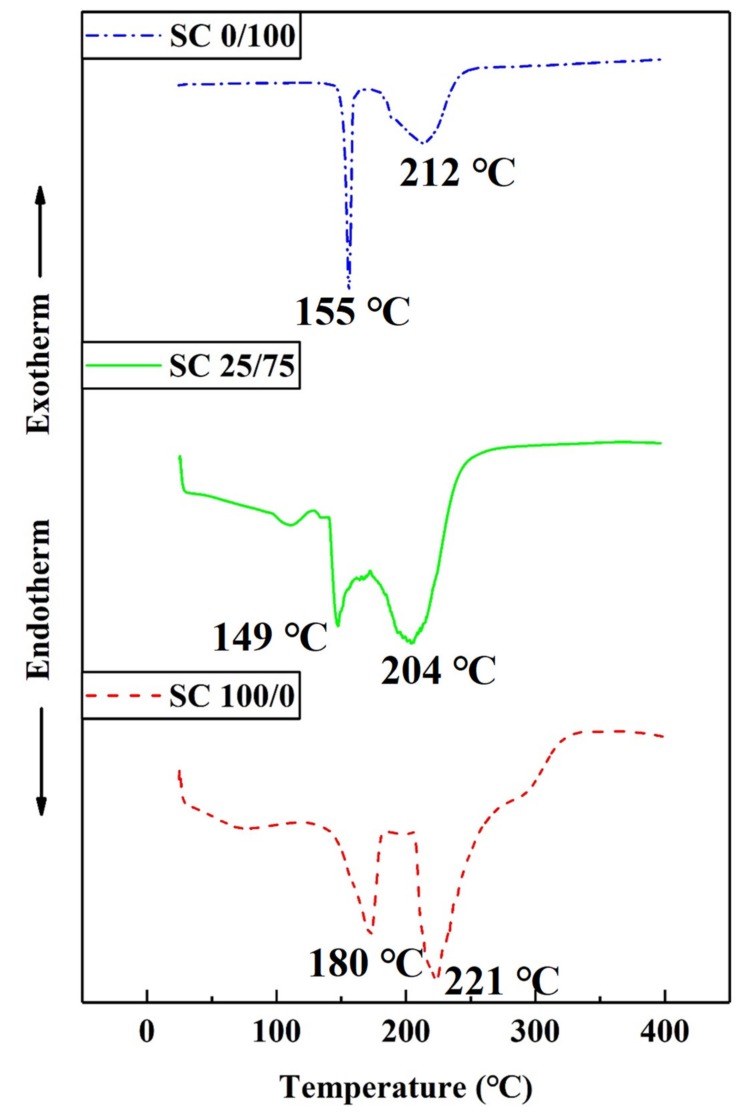
DSC curves of sucrose (SC 100/0), citric acid (SC 0/100) and uncured SC adhesive (SC 25/75).

Figure 5 shows the DSC curves of SC 100/0, 25/75, and 0/100. The DSC trace of SC 100/0 displayed two endothermic peaks at 180 and 221 °C. Based on the TG analysis, these temperatures were lower than the initial rapid mass loss temperature, and these peaks were attributed to the melting and caramelization of sucrose, respectively [26,29]. For citric acid, a sharp endotherm was observed at 155 °C, as well as a broad endotherm near 212 °C, which were, respectively, due to the fact of melting and decomposition [27]. The DSC curve of SC 25/75 showed that the two endotherm peaks shifted to lower temperatures (149 and 204 °C). The TG analysis showed that, although 149 °C was higher than the initial mass loss temperature of SC, combined with the DTG curve, rapid mass loss occurred near 194 °C; therefore, the endothermic peak located at 149 °C was most likely due to the melting of freeze-dried, uncured SC adhesive. Another broad peak at 204 °C was very close to the characteristic peaks of sucrose and citric acid, and it was difficult to attribute using only DSC; however, it can be speculated that it was due to the decomposition of citric acid, the degradation of sucrose or the reaction between sucrose and citric acid.

#### 3.2.3. Insoluble Mass Proportion

**Figure 6:**
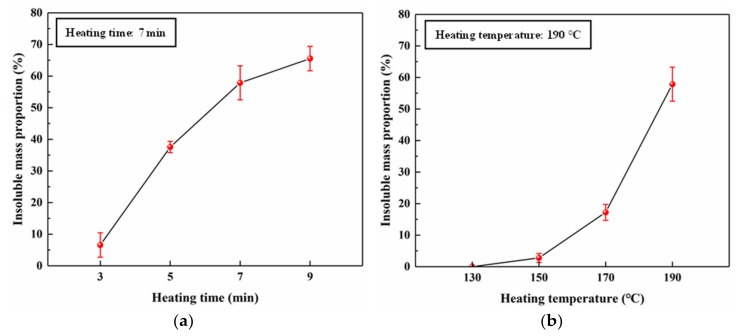
Effects of (**a**) heating time and (**b**) heating temperature on the insoluble mass proportion.

To investigate the relationship between the heating conditions and curing behaviour of SC 25/75, insoluble mass proportion measurements were carried out, and the results are shown in Figure 6. Figure 6a shows that increasing the heating temperature increased the insoluble mass proportion, indicating a positive correlation between temperature and curing of the SC adhesive, and the highest value (58%) was obtained at 190 °C. When the heating temperature was less than 170 °C, the insoluble mass proportion was less than 20%. This was due to the insufficient curing of the SC adhesive as shown by the absence of a rapid mass loss or endothermic peak in the thermal analysis at 170 °C. In addition, compared with the insoluble mass proportion obtained at 170 °C (17%), a pronounced increase was observed at 190 °C which implied that the DTG mass loss peak and endothermic DSC peak of SC 25/75 were most likely due to the curing between citric acid and sucrose. Based on the board property evaluation, the insoluble mass obtained at 190 °C increased the water resistance of the plywood. Figure 6b shows a nearly linear increase in the insoluble mass proportion as the heating time increased. It is worth noting that this trend was very similar to the results obtained from the plywood shear strength bonded with various hot-pressing times in Figure 2, indicating that the bonding performance of the SC adhesive was positively correlated with the insoluble mass proportion.

#### 3.2.4. Py-GC/MS

**Figure 7:**
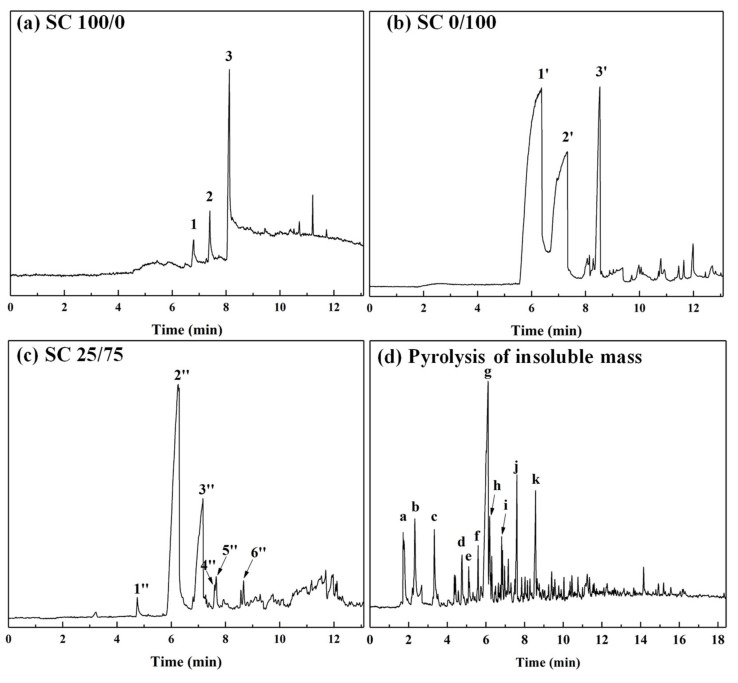
GC/MS chromatogram of the evolved gas derived from SC adhesives: (**a**) SC 100/0, (**b**) SC 0/100, (**c**) SC 25/75 heated at 170 °C for 60 s, (**d**) insoluble mass derived from cured SC 25/75 adhesive and heated at 500 °C for 60 s.

The Py-GC/MS chromatograms were obtained to investigate the chemical reactions during the curing process and the chemical composition of uncured SC 100/0, 0/100 and 25/75 adhesives and the cured insoluble mass of SC 25/75. Figure 7a–c show the GC/MS chromatograms of evolved gas obtained by heating the uncured adhesives at 190 °C. The identified volatile components with the highest and >80 similarity index (SI) were recorded in Table 2. The pyrogram of SC 100/0 (sucrose) contained three main peaks which were identified as 2,5-furandicarboxaldehyde (DFF), 4H-pyran-4-one,2,3-dihydro-3,5-dihydroxy-6-methyl- (DDMP), and 5-hydrxoymethylfurfural (5-HMF) which are characteristic pyrolysis products obtained by heating saccharides [30,31,32,33]. The primary product peaks marked 1’ and 2’ of SC 0/100 were attributed to citraconic acid anhydride and itaconic anhydride which were formed during the dehydration and decarboxylation of citric acid [34]. In addition, another obvious peak marked by 3’ was observed in the chromatogram of SC 0/100. Although this peak was identified as 5-HMF with a 95 similarity index, no published reports have shown the production of 5-HMF from the pyrolysis of citric acid. Considering the formation mechanism of 5-HMF [35], it is difficult to confidently assign this peak; therefore, it was labelled as “unknown” in this study.

Figure 7c shows the Py-GC/MS chromatogram of SC 25/75 which contains two noticeable peaks (marked 2” and 3”) attributed to citraconic acid anhydride and itaconic anhydride and which were produced during the pyrolysis of citric acid. The peaks labelled as 1”, 4”, and 5” were assigned to furfural, levoglucosenone (LGO), and maltol which formed during the degradation of sucrose [36,37]. According to the results in Figure 7a, these compounds were found in the synthesized SC 25/75 adhesive, suggesting that they formed due to the citric acid catalysing the decomposition of sucrose during heating. The peak marked 6” had a similar retention time as peak 3’ in Figure 7b and was due to the presence of an unknown compound. In addition, 5-HMF, a common degradation product of sucrose, was not observed in the chromatogram of SC 25/75, indicating this compound was possibly consumed during curing.

**Figure 8:**
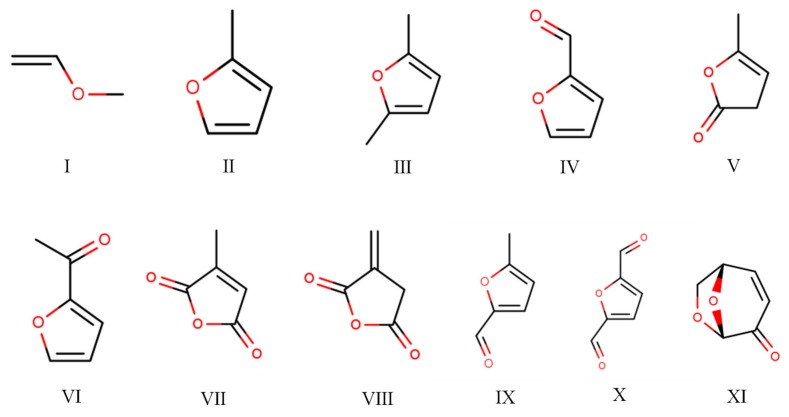
Chemical structure of the identified chemical compounds in evolved gas derived from insoluble mass of cured SC adhesives heated at 500 °C for 60 s.

**Figure 9:**
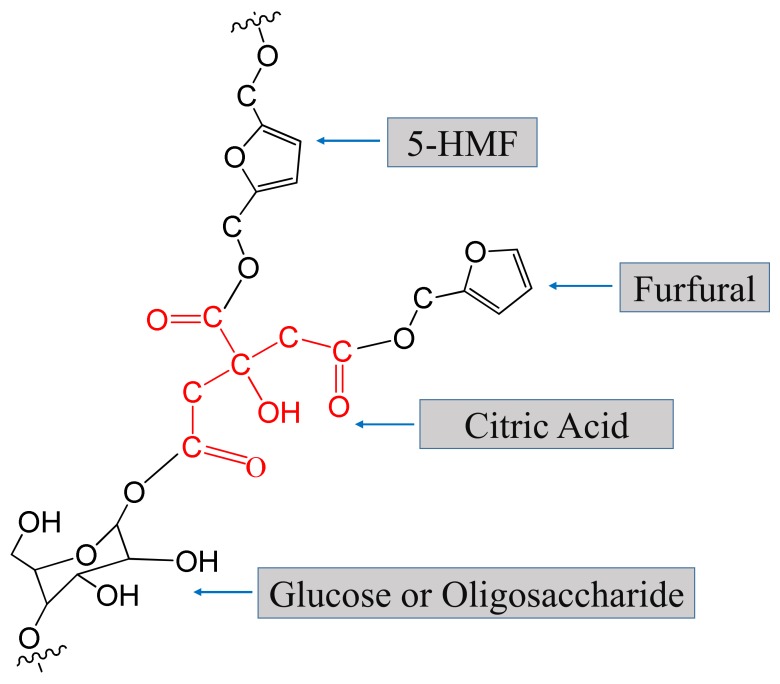
Part of the possible chemical structure of the cured SC adhesive.

To determine the chemical composition of the cured SC adhesives, the insoluble mass derived from SC 25/75 (heated at 190 °C for 7 min) was pyrolyzed at 500 °C for 60 s, and the Py-GC/MS chromatogram is shown in Figure 7d; the information and chemical structure of the identified compounds are listed in Table 3 and Figure 8. The peak located at 1.72 min (substance I: methoxyethene) was considered as an evidence of the formation of ether linkage in the cured adhesive, and this result has also been demonstrated by previous research [25]. In addition, it could be found that most of other chemical substances were furan compounds. The substances III, V, IX and X indicated that 5-HMF participated in the curing reaction, and other furan compounds with one substituent group (marked with II, IV, VI) were considered as the reaction between furfural and citric acid/5-HMF. The pyrolysis products of citric acid (citraconic acid anhydride and itaconic anhydride) were also detected in the insoluble mass which suggested that citric acid participated in the curing reaction. It is worth noting that levoglucosenone (marked with XI), which is usually formed by heating glucose or oligosaccharide, was also observed in the insoluble mass; this indicated the saccharide possibly participated in the curing reaction [38]. Based on the results of the Py-GC/MS above, a possible chemical structure of part of the cured SC adhesive is shown in Figure 9. The curing reaction of the SC adhesive can be described as a dehydration reaction occurring among citric acid, 5-HMF, glucose/oligosaccharide, and furfural, and a cross-link structure polymer was formed linked by C–O–C.

## 4. Conclusions

Sucrose and citric acid were utilized to synthesize an eco-friendly plywood adhesive (SC adhesive), and the effects of hot-pressing conditions on the bond performance were investigated. The results of the dry and wet shear strength tests showed that the optimal hot-pressing temperature, hot-pressing time, and spread rate of the SC adhesives were 190 °C, 7 min, and 140 g/m^2^, respectively. When plywood was bonded at these optimal hot-pressing conditions, the wet shear strength met the value specified by the China National Standard GB/T 9846-2015. The curing behaviour of the SC adhesive was studied by thermal analysis and insoluble mass proportion. Compared with sucrose and citric acid individually, the TGA and DSC curves of SC 25/75 showed lower thermal degradation and endothermic reaction temperatures. In addition, the insoluble mass proportion increased with the hot-pressing temperature and time. To further clarify the chemical transformation during curing and the chemical composition of the cured adhesives, Py-GC/MS analysis was carried out. The Py-GC/MS chromatograms showed that SC 25/75 produced volatile compounds, due to the decomposition of sucrose, which were not produced during the pyrolysis of SC 100/0. This indicated that citric acid-catalyzed the thermal degradation of sucrose. Furthermore, the main pyrolysis products of sucrose (5-HMF) were not observed during the heating of SC 25/75, implying that this compound was possibly consumed during curing. The Py-GC/MS results of the insoluble mass of cured SC 25/75 confirmed that SC 25/75 was cured by the reaction between furan compounds, citric acid, and glucose/oligosaccharide which produced an insoluble polymer with ether linkages. This study investigated the curing conditions and behaviours of synthesized SC adhesives and confirmed the chemical structure of the cured adhesive. Based on the synthesis and curing mechanism, methods to reduce the hot-pressing temperature and time will be studied in future research.

## Figures and Tables

**Table 1:** Manufacture conditions of the plywood bonded by the sucrose–citric acid (SC) adhesive.

Groups	Hot-Pressing Temperature (°C)	Hot-Pressing Time (min)	Spread Tate (g/m^2^)
	130		
**Group 1**	150	7	140
	170		
	190		
		3	
**Group 2**	190	5	140
		7	
		9	
			100
**Group 3**	190	7	120
			140
			160

**Table 2:** Identified chemical compounds in gas evolved from uncured SC adhesives heated at 190 °C for 60 s.

Samples	Peak Number	RT (min)	SI	Compound	CAS	MW	Formula
SC 100/0	1	6.79	93	2,5-Furandicarboxaldehyde	823-82-5	124	C_6_H_4_O_3_
	2	7.39	87	2,3-Dihydro-3,5-dihydroxy-6-methyl-4(4H)-pyranone	28564-83-2	144	C_6_H_8_O_4_
	3	8.22	94	5-Hydrxoymethylfurfura	67-47-0	126	C_6_H_6_O_3_
SC 0/100	1’	6.23	97	Citraconic acid anhydride or Itaconic anhydride	616-02-4 or 2170-03-8	112	C_5_H_4_O_3_
	2’	7.26	97	Citraconic acid anhydride or Itaconic anhydride	616-02-4 or 2170-03-8	112	C_5_H_4_O_3_
	3’	8.51		Unknown			
SC 25/75	1”	4.77	94	Furfural	98-01-1	96	C_5_H_4_O_2_
	2”	6.25	97	Citraconic acid anhydride or Itaconic anhydride	616-02-4 or 2170-03-8	112	C_5_H_4_O_3_
	3”	7.13	97	Citraconic acid anhydride or Itaconic anhydride	616-02-4 or 2170-03-8	112	C_5_H_4_O_3_
	4”	7.62	81	Levoglucosenone	37112-31-5	126	C_6_H_6_O_3_
	5”	7.66	80	Maltol	118-71-8	126	C_6_H_6_O_3_
	6”	8.58		Unknown			

**Table 3:** Identified chemical compounds in evolved gas derived from insoluble mass of cured SC adhesives heated at 500 °C for 60 s.

Peak Number	RT (min)	SI	Compound	CAS	MW	Formula	Chemical Structure Number
a	1.72	96	Methoxyethene	107-25-5	58	C_3_H_6_O	I
b	2.32	94	2-Methylfuran	534-22-5	82	C_5_H_6_O	II
c	3.33	96	2,5-Dimethylfuran	625-86-5	96	C_6_H_8_O	III
d	4.77	94	Furfural	98-01-1	96	C_5_H_4_O_2_	IV
e	5.15	90	2,3-Dihydro-5-methyl-2-furanone	591-12-8	98	C_5_H_6_O_2_	V
f	5.59	96	2-Acetylfuran	1192-62-7	110	C_6_H_6_O_2_	VI
g	6.01	97	Citraconic acid anhydride or Itaconic anhydride	616-02-4 or 2170-03-8	112	C_5_H_4_O_3_	VII VIII
h	6.11	94	5-Methylfurfural	620-02-0	110	C_6_H_6_O_2_	IX
i	6.82	91	2,5-Furandicarboxaldehyde	823-82-5	124	C_6_H_4_O_3_	X
j	7.60		Levoglucosenone	37112-31-5	126	C_6_H_6_O_3_	XI
k	8.60		Unknown				
